# Vitamin D deficiency and associated factors among antenatal care attending pregnant women in Sodo town, South Ethiopia: A facility-based cross-sectional study

**DOI:** 10.1371/journal.pone.0279975

**Published:** 2022-12-30

**Authors:** Dibora Teferi Haile, Takele Tadesse Damote, Fikadu Elias Sadamo, Zeleke Geto Demissie, Samson Kastro Dake

**Affiliations:** 1 Department of Reproductive Health and Nutrition, College of Health Sciences and Medicine, School of Public Health, Wolaita Sodo University, Wolaita Sodo, Ethiopia; 2 Department of Biostatistics and Epidemiology, College of Health Sciences and Medicine, School of Public Health, Wolaita Sodo, Ethiopia; 3 National References Laboratory for Clinical Chemistry, Ethiopian Public Health Institute, Addis Ababa, Ethiopia; Madda Walabu University, ETHIOPIA

## Abstract

**Background:**

Vitamin D deficiency is an emerging public health problem globally, with devastating health consequences. Pregnant women are most susceptible for Vitamin D deficiency, and black women particularly are under double burden of the problem. Therefore, this study aimed to determine the prevalence of Vitamin D deficiency and identify associated factors among antenatal care attending pregnant women.

**Methods:**

A facility-based cross-sectional study involving 331 pregnant women was conducted from March to April in 2021. Systematic random sampling technique was used to select the study participants from antenatal care service providing facilities. Data were collected by using interviewer-administered questionnaire and 5ml of blood sample was collected using aseptic techniques. Data were entered into Epi Data software version 3.1 and exported to SPSS version 20 for analysis. Binary logistic regression analysis was used to identify the associated factors.

**Results:**

In this study, about 39% of the women were Vitamin D deficient; of which 8.8% were severely deficient. The mean serum Vitamin D level was 24.43ng/ml. Women with Body Mass Index (BMI) ≥30 (AOR = 47.31; 95% CI: 3.94, 567.70) and who never ate egg had a higher chance of being Vitamin D deficient (AOR = 7.48; 95% CI: 1.02, 55.05). On the other hand, women who were exposed to mid-day time sunlight (AOR = 0.30; 95% CI: 0.11, 0.77) were less likely to become Vitamin D deficient.

**Conclusions:**

Vitamin D deficiency is higher among obese women and women who did not consume egg. Being exposed to mid-day sunlight is protective against Vitamin D deficiency. Having optimal body weight, mid-day sun light exposure and consumption of Vitamin D rich diet might contribute to reduce the risk of Vitamin D deficiency.

## Introduction

Vitamin D is a fat-soluble vitamin that can be found in two forms Vitamin D3 (Cholecalciferol) and Vitamin D2 (Ergocalciferol) [1]. Vitamin D3 is produced in the skin in response to ultraviolet B (UVB) radiation or from the animal source diet. Vitamin D2 is found in some plants in the diet and is produced commercially by irradiation of yeast [2]. The best indicator of an individual’s vitamin D status is 25-hydroxycholecalciferol [25(OH) D], which reflects both cutaneous synthesis and dietary consumption of the nutrient [3].

About 80% - 90% source of vitamin D is sunlight, and Vitamin D in a diet is essential when the UVB radiation is not sufficient [1]. However, UVB radiation can be affected by water vapors, air pollution, and time of day, solar zenith angle, season and latitude. Clothing, Sunscreen, skin pigment, burnt scar, body fat, age and skin temperature can also interfere with UVB–7-dehydrocholesterol interactions by absorbing UVB radiation [4]. Anti-TB and anti-epileptic medications negatively affect serum vitamin D level [5]. Since liver and kidney are the main organs acting on the synthesis of active form of vitamin D, diseases related with these organs highly affect vitamin D status [6].

Vitamin D status has been linked to chronic diseases like cancer, cardiovascular disease, autoimmune disease and infection [7]. Vitamin D is important for growth and development, including regulation of cellular differentiation and apoptosis immune system development, and brain development [8–10].

Vitamin D deficiency is related with pregnancy complications such as preeclampsia, gestational diabetes, cesarean section, preterm birth, vaginal infections, intrauterine growth restriction, and spontaneous abortion [11–16]. Vitamin D deficiency is incipient public health problem and more than one billion people from all age group and ethnics are deficient [17]. It has been gaining much attention globally due to its health effects on the reduction of mortality and morbidity [18].

Vitamin D deficiency is common in Australia, the Middle East, India, Africa, and South America [19]. There is a significant variation in the prevalence of Vitamin D deficiency globally and the prevalence ranges between 10.1% in Denmark and 84.2% in Ethiopia [11,20,21].

In Ethiopia vitamin D rich foods are rarely consumed and there is no fortification program [22,23]. Dark-skinned people are particularly subject to deficiency without adequate sunlight exposure or dietary intake [22].

Pregnant women are more susceptible to vitamin D deficiency than any other human group [24]. Vitamin D deficiency has potentially adverse consequences for the women’s own health and has inter-generational effect on the fetus [22]. Although Ethiopia is one of the sunshine rich countries, vitamin D deficiency is most prevalent in school-age children [23] as well as in the reproductive age group women [22]. As to the investigators knowledge, there is paucity of data on the prevalence of vitamin D deficiency among pregnant women in Ethiopia. The Ethiopian Minister of Health identified vitamin D deficiency as an emerging public health problem and recommended other studies to be carried out in the area [25]. Thus, this study aimed at determining the prevalence of Vitamin D deficiency and identifying the factors associated with it among antenatal care (ANC) attending pregnant women in South Ethiopia.

## Methods and materials

### Study design, area and period

After receiving ethical letter from "Hawassa University Medicine and Health Science Institutional Review Board by reference number IRB/155/10, a facility-based cross-sectional study was conducted from March to April 2021 in Wolaita Sodo town. The board considered respect for person, beneficence and Justice. Written consent was obtained from participants". Wolaita Sodo town is located at 327 km Southwest of Addis Ababa. The town is situated at a latitude and longitude of 60 54’N 370 45’E with an elevation between 1,600 and 2,100 meters above sea level. It has a census projected population of 270,764; 136,762 being women in reproductive age group. One public hospital, 1 private hospital, 3 health centers, and 8 other private clinics deliver ANC service to the population in the town. There were an estimated 8,936 pregnant women in 2021 in the town.

### Population and sampling

The source populations for this study were all pregnant women in the town. The study populations were randomly selected ANC attending first-trimester pregnant women who have ANC follow up in the selected health facilities. Women with confirmed kidney disease, liver disease, having major burn scar, on anti-TB, on epileptic medications and severely ill during data collection were excluded from the study. A single population proportion formula with the following assumptions was used to calculate a sample size of 348; 95% confidence level, 5% margin of error, an estimated prevalence of Vitamin D deficiency of 29% [26], and 10% non-response rate. The sample size was allocated to the selected 2 hospitals, 3 health centers and 5 private clinics proportional to the number of ANC attendants in the previous year in each facility. Systematic random sampling was used to select the study participants.

## Variables

### Outcome variable

Serum 25 (OH) D levels of the pregnant women was an outcome measure. It was considered Vitamin D deficiency if the serum 25 (OH) D levels was less than 20ng/ml [3].

#### Exposure variables and covariates

Socio-demographic and economic factors (age, income, occupation, education, and ethnicity), dietary intake, anti-epileptic and anti-TB medications intake, presence of kidney and liver disease, body composition (BMI and skin pigment), maternal obstetric conditions (inter-pregnancy interval, gravidity, parity, and mode of delivery), smoking and alcohol consumption, sun light exposure modulators (seasons, sunlight exposure time of the day, utilization of sun screen, type of clothing).

Major burn: First or second-degree burn covering more than 25% of an adult’s total body surface area (TBSA) or third-degree burn on more than 10% TBSA. In addition, burns involving the hands, feet, and face [27].

Sunlight exposure time

Morning: The time period between 7:00AM– 9:00AM

Mid-day: The time period between 10:00AM– 3:00PM

Evening: The time period between 4:00PM– 6:00PM

### Data collection

Data were collected during the winter season by using interviewer administered structured questionnaire. BSc degree holder midwives who are proficient in the local language and with previous data collection experience conducted the interviews, and laboratory technologists collected the blood samples. Close supervision of the data collection was done by experts. Weight was measured by using a well calibrated portable digital flat Seca scale and height was measured by using portable stadiometer. All measurements were taken three times, and the average was recorded as the final measurement. Preconception weight adjustment was done before BMI calculation and compared to WHO references [28]. Gestational age of the women was calculated by using the date of the last normal menstrual period (LNMP). For participants whose LNMP was unknown, the ultrasound and fundal height were used to determine the gestational age. Skin pigmentation was classified based on Fitzpatrick skin type classification and study population skin colors were classified as type IV-VI; light brown, dark brown and very dark respectively.

A non-fasting venipuncture 5ml blood sample was collected from the antecubital vein of all participants in a dark room by using aseptic techniques and standard procedures. The serum had been separated by use-and-through plastic pipette and stored in refrigerator at data collection site for less than of 10 hours. Then, transported to Otona Teaching and Referral Hospital daily and had been stored in deep freeze (-20oc and above). Sample was covered by aluminum foil until analysis time. After completion of the data collection, the sample was transported during nighttime to EPHI laboratory by triple ice pack. The recommended maximum duration for sample storage in the deep freeze is six months whereas we stored for four months before the analysis [29]. Serum 25 (OH) D levels were tested at the Ethiopian Public Health Institute laboratory by Roche electro-chemo immunoassay diagnostic assay, Elects vitamin D total II assay kit on Cobas e 411 immunoassay analyzer.

### Data analysis

Then data were entered into Epi Data version 3.1 and analyzed by using SPSS software version 20. Descriptive statistics were done for the main variables. Bivariate logistic regression analysis was used to select exposure variables with a crude association to the outcome. All exposure variables with *p*-value less than 0.25 during bivariate analysis were taken for multivariate analysis. Finally, multivariate analysis was done to control for potential confounders and identify independent predictors of the outcome. Normality of all continues variables was checked by using Kolmogorov-Smirnov test at p-value >0.05. Adjusted odds ratio along with 95% CI was estimated to measure the strength of association, and statistical significance was declared at *p*-value less than 0.05.

## Result

### Socio-demographic and economic characteristics

Overall, 331 pregnant women participated in this study, making the response rate 95.1%. The mean age of the participants was 24.7 ± 4.9 years. More than two third, 223 (67.4%) attended secondary education and above, and 176 (53.2%) were housewives. The majority, 322 (97.3%) of the women were married and 66 (19.9%) of the households were in the lowest wealth index (Table 1).

**Table 1:** Socio-demographic and economic characteristics of the study participants in Sodo town, South Ethiopia, March 2021.

Variables	Category	Frequency	Percent
Age	18–25	215	65
	26–49	116	35
Educational status	non formal education	30	23.5
	Primary	78	9.1
	Secondary	126	38.1
	Above secondary	97	29.3
Occupation	Housewife	176	53.2
	Merchant	56	16.9
	Government/ NGO	75	22.7
	Student/ daily laborer	24	7.3
Religion	Protestant	198	59.8
	Orthodox	94	28.4
	Others*	39	11.8
Marital status	Married/living together	322	97.3
	Divorce/separated	5	1.5
	Widowed	2	.6
	Not married	2	.6
Husband education	No formal education	16	4.8
	Primary	71	21.5
	Secondary	110	33.3
	Above secondary	133	40.2
Family size	One–four	276	83.4
	Five and above	55	16.6
Wealth index (quintile)	Lowest	66	19.9
	Second	66	19.9
	Middle	70	21.1
	Fourth	64	19.3
	Highest	65	19.6
Housing condition	Basement	325	98.2
	Ground plus one & above	6	1.8

*Catholic, and Muslim.

### Maternal obstetric characteristics and body composition

The mean gestational age of the participants was 8.4 ± 2.9 weeks. About 148 (44.7%) of the women were primigravida and 89 (26.9%) were multiparous. More than three fourth, 139 (75.9%) of the participants who have given birth previously had an inter-pregnancy interval of two years or lesser. About nine in ten, 162 (88.5%) women gave birth through spontaneous vaginal delivery on their previous birth. Regarding skin color scoring, 155 (46.8%) had dark brown (type V) skin whereas 102 (30.8%) had very dark brown (type VI) skin. The majority, 240 (72.5%) of the participants had their BMI under the normal range and 17 (5.1%) were obese (Table 2).

**Table 2:** Maternal obstetric characteristics and body composition of the study participants at Sodo town in South Ethiopia, March 2021.

Variables	Category	Frequency	Percent
Gravidity(Number of pregnancy)	Primigravida	148	44.7
	Multigravida	183	55.3
Parity(number of delivery)	Nulliparous	148	44.7
	Primiparous	94	28.4
	Multiparous	89	26.9
Inter pregnancy interval	≤ 2 year	44	24.1
	> 2 years	139	75.9
Mode of delivery of previous pregnancy	Vaginal delivery	162	88.5
	Caesarian section	21	115
BMI	Under weight(<18.5)	16	4.8
	Normal(18.5–25)	240	72.5
	Over weight(>25–30)	58	17.5
	Obese(>30)	17	5.1
Skin color	Type IV	74	22.4
	Type V	155	46.8
	Type VI	102	30.8

### Dietary intake of Vitamin D rich foods

About 197 (59.5%) of the women never consumed fish and 317 (95.8%) never consumed vitamin D fortified cereals. On the other hand, 150 (45.3%) consumed egg at list 1–3 times in a week or more (Table 3).

**Table 3:** Dietary intake of Vitamin D rich or fortified food by the study participants at Sodo town in South Ethiopia, March 2021.

Variables	Category	Frequency	Percent
Fish	4–6 times per week	2	0.6
	1-3times per week	6	1.8
	1–3 times per month	20	6
	occasional/in holy days	106	32
	Never	197	59.5
Fish oil	Ever consumed	11	3.3
	Never	320	96.7
Mushroom	Ever consumed	22	6.6
	Never	309	93.4
Fortified milk and milk products	Ever consumed	25	7.6
	Never	306	92.4
Egg	4–6 times per week	46	13.9
	1-3times per week	104	31.4
	1–3 times per month	107	32.3
	occasional/in holy days	46	13.9
	Never	28	8.5
Fortified oil	4–6 times per week	13	3.9
	1-3times per week	4	1.2
	1–3 times per month	5	1.5
	occasional/in holy days	18	5.4
	Never	291	87.9
Liver	1-3times per week	34	10.3
	1–3 times per month	84	25.4
	occasional/in holy days	76	23.1
	Never	137	41.4
Vitamin D fortified cereals	Ever Consumed	**14**	**4.2**
	Never	**317**	**95.8**
Cake and confectionary	4–6 times per week	4	1.2
	1-3times per week	18	5.4
	1–3 times per month	39	11.8
	occasional/in holy days	79	23.9
	Never	191	57.5
Vitamin D supplements	occasional/in holy days	4	1.2
	Never	327	98.8

### Sunlight exposure and lifestyle characteristics

About 318 (96.1%) of the women never used sunscreen and 269 (81.3%) use an umbrella. About 188 (56.8%) of the participants reported walking as an outdoor activity. About 105 (31.7%) of the participants were exposed to sunlight in the morning whereas 124 (37.5%) mid-day. Regarding the duration of sunlight exposure, 197 (59.5%) of the women were exposed for over 30 minutes. The majority, 293 (88.5%) of the study participants expose >15% of their TBSA for sunlight (Table 4).

**Table 4:** Sunlight exposure and life style characteristics of the study participants at Sodo town in South Ethiopia, March 2021.

Variables	Category	Frequency	Percent
Sunscreen lotion use	Ever used	13	3.9%
	Never	318	96.1%
Umbrella	Sometimes	156	58
	Often	85	31.6
	Always	28	10.4
Taboos practice	No	290	87.6
	Yes*	41	12.4
Alcohol drinking	No	324	97.9
	Yes	7	2.1
Smoking history	No	324	97.9
	Yes	7	2.1
Outdoor activity	Marketing	111	33.5
	Walking	188	56.8
	No activity	20	6
	Washing cloth	12	3.7
Day time of sun exposure	Morning	105	31.7
	Mid-day	124	37.5
	Evening	82	24.8
Duration of sun exposure	<30 minuet	134	40.5
	≥30-1hour	143	43.2
	>1 hour	54	16.3
Total body surface area exposed	<15% of TBSA	38	11.5
	15–20% of TBSA	183	55.3
	>20–30% of TBSA	52	15.7
	>30% of TBSA	58	17.5

*Types of taboos restricted were tight cloth, kasaba, Milk, Adenguare, Godare, fruit and cabbage.

### Vitamin D level of pregnant women

The mean serum Vitamin D level was 24.43ng/ml ± 11.34ng/ml. The prevalence of Vitamin D deficiency in this study was 39.0% (Table 5).

**Table 5:** Serum Vitamin D level of the study participants at Sodo town in South Ethiopia, March 2021.

Total serum vitamin D level	Frequency	Percent	Cumulative percent
Below detection level (< 3 ng/ml)	4	1.2	1.2
Sever deficiency (3 ng/ml—>10 ng/ml)	25	7.6	8.8
Deficiency (< 20 ng/ml)	100	30.2	39.0
Insufficient (≥20 ng/ml—30 ng/ml)	108	32.6	71.6
Sufficient (>30 ng/ml)	94	28.4	100.0
Total	331	100	

### Factors associated with Vitamin D deficiency

Women with BMI ≥30 and had a 47 times higher chance of becoming Vitamin D deficient (AOR = 47.31; 95% CI: 3.94, 567.70). Women who never consumed egg had about 8 times higher chance of becoming Vitamin D deficient (AOR = 7.48; 95% CI:1.02, 55.05). On the other hand, women who were exposed to mid-day time sunlight had 70.0% lesser chance of having Vitamin D deficiency (AOR = 0.30; 95% CI: 0.11, 0.77) (Table 6).

**Table 6:** Factors associated with Vitamin D deficiency among the study participants at Sodo town in South Ethiopia, March 2021.

Variables	Category	Serum level <30ng/ml	Serum level ≥30ng/ml	COR (95% CI)	AOR (95% CI)
**Age**	18–25	81(37.7%)	134(62.3%)	1	
	26–49	48(41.4%)	68(58.6%)	0.86(0.54, 1.36)	1.02(0.42, 2.45)
**Occupation**	House wife	69(39.2%)	107(60.8%)	1	
	Merchant	20(35.7%)	36(64.3%)	1.16(0.62, 2.17)	1.28(0.49, 3.34)
	Government/NGO	27(36%)	48(64%)	1.15(0.66, 2.01)	1.27(0.43, 3.80)
	Student/laborer	13(54.2)	11(45.8)	0.55(0.23, 1.29)	1.55(0.25, 9.70)
**Educational status**	Primary and below	47(43.5%)	61(56.5%)	1	
	Secondary	49(38.9%)	77(61.1%)	1.21(0.72, 2.04)	0.53(0.20, 1.39)
	Above secondary	33(34%)	64(66%)	1.49(0.85, 2.63)	0.41(0.11, 1.53)
**Birth interval**	≤ 2 years	17 (38.6%)	27 (61.4%)	1	
	> 2 years	55 (39.6%)	84(60.4%)	0.96(0.48, 1.93)	1.07(0.45, 2.53)
**N****o** **of birth**	Primiparous	32(34%)	62(66%)	1	
	Multiparous	40(44.9%)	49 (55.1%)	0.77(0.45, 1.31)	1.54(0.68, 3.46)
**Umbrella**	Yes	99 (36.8%)	170(63.2%)	1	
	No	30(48.4%)	32 (51.6%)	0.62(0.36, 1.08)	1.08(0.41, 2.79)
**Mode of delivery**	SVD	62(38.3%)	100(61.7%)	1	
	CS	10(47.6%)	11(52.4%)	0.68(0.27, 1.70)	3.20(.92, 11.11)
**BMI**	<18.5	4(25%)	12(75%)	1	
	18.5–25	83(34.6%)	157(65.4%)	0.63(0.20, 2.02)	4.06(0.66, 24.97)
	>25–30	28(48.3%)	30(51.7%)	0.36(0.10, 1.24)	6.70(0.94, 47.57)
	> 30	14(82.4%)	3(17.6%)	0.07(0.01, 0.39)	47.31(3.94, 567.70)
**TBSA**	<15%	23(39%)	36(61%)	1	
	≥15%	106(39%)	166(61%)	1.01(0.56, 1.78)	0.99(0.39, 2.50)
**Egg consumption**	Per day	18(39.1%)	28(60.9%)	1	
	Per week	40(38.5%)	64(61.5%)	1.03(0.51, 2.10)	3.38(0.82, 13.96)
	Per month	42(39.3%)	65(60.7%)	0.99(0.49, 2.02)	3.47(0.77, 15.59)
	Occasional	18 (39.1%)	28(60.9%)	1.00(0.43, 2.31)	5.14(0.90, 29.32)
	Never	11(39.3%)	17(60.7%)	0.99(0.38, 2.60)	7.48(1.02, 55.05)
**Liver consumption**	Per week	6(17.6%)	28 (82.4%)	1	
	Per month	40(47.6%)	44(52.4%)	2.20(0.19, 25.20)	2.59(0.61, 10.99)
	Occasional	29(39.7%)	44(60.3%)	3.03(0.26, 35.02)	1.28(0.27, 6.04)
	Never	52(38%)	85(62%)	3.27(0.29, 36.95)	0.97(0.22, 4.29)
**Time of sunlight exposure**	Morning	45(42.5%)	60(57.1%)	1	
	Mid-day	47(37.9%)	77(62.1%)	1.23(0.72, 2.09)	0.30(0.11, 0.77)
	Evening	33(40.2%)	49(59.8%)	1.11(0.62, 2.00)	0.57(0.22, 1.50)
**Duration of sunlight exposure**	≤ 30min	50(37.3%)	84(62.7%)	1	
	30-60min	61(42.7%)	82(57.3%)	0.80(0.49, 1.30)	1.92(0.84, 4.42)
	>1hr	18(33.3%)	36(66.7%)	1.19(0.61, 2.32)	1.07(0.33, 3.49)
**Wealth index**	Lowest	25(37.9%)	41(62.1%)	1	
	Second	28(42.4%)	38(57.6%)	1.09(0.54, 2.21)	1.397(.44, 4.40)
	Middle	28(40%)	42(65.6)	0.91(0.45, 1.82)	1.64(0.50, 5.41)
	Fourth	22(34.4%)	42 (60%)	1.00(0.50, 1.99)	1.59(0.42, 5.94)
	Highest	26(40%)	39(30%)	1.27(0.62, 2.60)	1.63(0.39, 6.79)
**Religion**	Protestant	76(38.4%)	122(61.6%)	1	
	Orthodox	36(38.3%)	58(61.7%)	1.01(0.61, 1.66)	0.56(0.24, 1.33)
	Others	17(43.6%)	22(56.4%)	0.81(.402–1.615)	0.75(0.23, 2.41)

## Discussion

In this study, the prevalence of vitamin D deficiency was reported to be 39.0%. Studies conducted in Nigeria and Turkey reported a prevalence level of 29% and 45.9% respectively, which are comparable with the current study [26,30].

Contrary to this finding, a study conducted in central Ethiopia reported a higher prevalence level (81%) [31]. This difference might be due to the small sample size, the sampling technique utilized and the data collection season (rainy season) in the above mentioned study. Another study conducted among reproductive-age women in South Ethiopia reported a prevalence level of 84.2% [22]. A higher prevalence reported in this study could be explained by the high fluoride content of the lakes and ground water in Rift valley, which has an association with hypocalcaemia and rickets [32]. Another study from Morocco also reported a higher prevalence (90.1%) [33]. Morocco is geographically located in a region where there is a reduced sunlight radiation. A slightly higher prevalence was reported by a study from Saudi Arabia where the prevalence of Vitamin D deficiency was 50% [34]. This variation might be due to the clothing style of the women that covers face and feet. Covering face and feet outdoor can protect sunlight exposure and result in vitamin D deficiency [35].

In this study women who have never consumed egg were at an increased risk of vitamin D deficiency. In consistent with this study finding, a study conducted in south Ethiopia reported that Vitamin D rich foods were rarely consumed [22]. Vitamin D rich foods like mushroom, fish, fish oil, fortified cereals, milk and milk products, other fortified foods and supplementations were almost never consumed. This might have contributed for the insufficiency of vitamin D [35]. This is supported by a study in Saudi Arabia where changes in dietary habit to the consumption of fast foods at the expense of nutrients-dense foods was associated with Vitamin D deficiency [34]. Another study conducted in New Zealand indicated no significant association between dietary intake and serum Vitamin D level [36].

In this study, mid-day time sunlight exposure was found to be protective against vitamin D deficiency. The study area is situated at the latitude of 60 54’N with an elevation between 1,600 and 2,100 meters above sea level. Even though Ethiopia is one of a country with abundant sunlight throughout the year and, this study vitamin D status reviles contradiction that might be in line with Saudi study whose clothing style was dark and restricted daytime activity [34]. The study population dressing style of any religious group did not cover the face, hand, and feet.

Only 7(2.1%) women had a smoking history. The hypovitaminosis D rate from smokers was 86% (n = 6). However, smoking is not retained as a significant factor in this study, perhaps it might be due to the very low prevalence of smokers. A study conducted in Belgium revealed that the risk of vitamin deficiency increases on smokers than non-smokers [37]. The possible reason for this is unknown.

BMI increment has shown a significant association with low level of serum vitamin D level in this study. This finding is in line with studies conducted in Belgium and China [37,38]. This might be because obesity results in sequestration of vitamin D in body fat which can reduce its availability [37]. However, a study from Morocco reported no significant association between Vitamin D level and body composition [33].

### Strength and limitations of the study

The main strength of this study is assessing the serum level vitamin D deficiency among the most at risk group, pregnant women in a well-organized laboratory. This study was limited to health facilities in scope and used a cross-sectional data. Therefore, it might not show the prevalence among those women who do not have ANC follow-up. In addition, we did not get a quantified measurement of UVB sunlight exposure for the study participants.

## Conclusions

This study ascertained that Vitamin D deficiency is higher among obese women and women who did not consume egg. It was also found that being exposed to mid-day sunlight is protective against Vitamin D deficiency. We recommend that all pregnant women should have an optimal pre-pregnancy body weight and get adequate mid-day sunlight exposure. We also recommend that pregnant women should consume egg and other Vitamin D source foods.

## Supporting information

S1 Dataset (SAV)
